# Wiley's automatic waiver of article processing charge (APC): Iterating contribution to the science from the underserved

**DOI:** 10.1002/ccr3.9471

**Published:** 2024-10-16

**Authors:** Prajwal Pudasaini, Charles Young

**Affiliations:** ^1^ Civil Service Hospital Ringgold Standard Institution—Dermatology Kathmandu Nepal; ^2^ Wiley—CCR, Atrium Chichester UK

## 1 INTRODUCTION

The transition to open access publishing has been instrumental in paving the way towards equitable access to global scientific information. This progression is clearly illustrated by the fact that 50% of scholarly articles are now published in open access journals.^1^ In addition, and emphasizing the importance of this transition, those articles published in open access titles are cited 50% more frequently than articles published behind subscription paywalls.^2^ Open access publishing then, is already having a dramatic impact both on what information is available to a global readership, and how that information is used.

However, open access publishing is not without challenges. The Article Publication Charge (APC) is the fee paid by the researcher, their institution, or their funder to publishers to contribute to the costs of making content open access. APCs in this context may limit the ability of some authors to share their research with readers, and so hinder the open access concept's central goal of providing equitable access to scholarly information—especially for authors working in Low‐ and Middle‐Income Countries (LMIC). With an aim of ensuring a more inclusive approach to research publication, some publishers provide waivers for APCs, based on criteria including per capita income, total Gross National Income, healthy life expectancy, and other country‐level and human development indicators.^3^

Wiley has a very well‐established APC Waiver Policy supporting research publication in around 125 countries, based on metrics including the human development index. In the context of this Policy, corresponding authors from LMIC countries receive either a full or 50% APC waiver based on Research4Life criteria.^4^ In 2022, the Wiley APC waiver Policy provided full or 50% waivers for a total of 1850 open access articles, amounting to a $4.6 million USD contribution to facilitate open access publishing in LMICs.^5^ Much harder to measure however, is the significant impact that Wiley's APC waiver Policy continues to make on enhancing the ability of authors from LMICs to share their research globally, and more broadly on facilitating scientific discovery and the advancement of global healthcare.

Clinical Case Reports (CCR), published by Wiley, is a leading, global, peer‐reviewed, gold open access biomedical journal publishing case reports, clinical images, and clinical videos. In 2023, there were over 2.3 million full‐text views of CCR publications. Along with its many well‐established and well‐published authors, CCR has always encouraged submissions from, and publications by, early‐career authors including those from LMICs. APC waivers for these early‐career authors are often determinant in their ability to publish their work. Dr. Prajwal Pudasaini is an early‐career dermatologist practicing in Nepal, a published author, and one of the CCR editorial team. The following discussion between Dr. Pudasaini and Dr. Charles Young, CCR Editor in Chief, highlights some of the key challenges facing early‐career authors from LMICs and the importance of APC waivers.

CY Can you tell me what your clinical role is and why you want to publish research?

PP I'm a clinical dermatologist, in the third year of my practice and I work in a government hospital which caters to patients from all over the country. It is a 132 bed, not‐for‐profit, hospital that was built by the Peoples' Republic of China (PRC) and handed over to Nepal government in 2008. The hospital is located in Kathmandu, the capital city of Nepal, amidst a large area with lush green natural surroundings. As it is a government‐run hospital it provides health care services at subsidized rates to the general public from across the country. Our outpatient departments (OPDs) are usually busy and chaotic with an enormous number of patients accommodated within our 2 busy OPD rooms. Most of the patients I see are of low middle class and middle‐class socio‐economic backgrounds, coming from rural terrains, mountains, hills, and the far‐off Terai region of the country. Oftentimes, we are faced with financial hindrances for diagnostics and therapy due to lack of a robust health care and insurance system in Nepal. More often than not, we have to be cautious about the tests ordered and cost of medicines to be prescribed as these have to be paid by patients out of their own pocket. I'm directly involved in examining, diagnosing, and treating the vast majority of skin and venereal diseases. As most of our patients are from an impoverished region, devoid of health insurance facilities, we have to be considerate of the cost–benefit and effectiveness of therapy whilst treating our patients.

In my view, medical science, including dermatology, should be an evidence‐based practice as far as possible, which relies on high quality research. Given the resource limitations, absence of research funding, and lack of government policies to encourage research, there is a scarcity of high‐quality practice guiding studies from our part of the world. Even in modern times, our practice is often still guided by research studies conducted in a different physical, socio‐cultural, ethnic, and racial environment to our own. Given the distinct phenotypic and genotypic characteristics of disease occurrence in patients from our region, we believe that local research publications will guide us by providing practical and locally relevant evidence. Furthermore, on an individual level, publications help us build our career prospects through scholarships and travel awards, which are mostly granted based on the number of publications one has made.

CY Why do you find it hard to publish your research?

PP I have always been fascinated by research and intrigued by the impact it can create for practicing clinicians and global scientists. However, for an early‐career clinician from a low‐income country, finding a secure job, financial stability, and looking after family become top priorities. Despite having a keen interest in research, much of our time is consumed by day‐to‐day hospital work and personal family responsibilities, and so our passion for research is often dimmed by these aforementioned tasks which take much of our days' time.

Research publication demands a noble idea, good write‐up, evidence, practicality, and usefulness in a local context. As early‐career researchers, we face setbacks in all of the above! Moreover, language barriers and negative bias during the editorial process are also some of the limiting factors that we face. Our creativity is often limited by the time and energy consumed through our day‐to‐day run from one to other clinic to see patients. Further, there are barely any research grants available, there is a lack of national policies which encourage research, lack of support from institutions, and a lack of capacity‐building training that promotes research. Moreover, as a clinician who works 6 days a week seeing almost 100 patients every day, I have the bare minimum of time and energy for creative writing, high‐quality research, or innovation.

CY Why do you want to publish case reports?

PP Case reports help my passion for research by allowing me to contribute to the scientific record through sharing clinical cases which are relevant in practice, and ultimately contribute to improving global health outcomes on a broader perspective. For someone with passion to write and publish but with time and financial and institutional limitations, case reports can be a vitally important tool. Although some see case reports at the base of evidence level pyramid, these reports directly help practicing clinicians tailor care based on excerpts and instances from similar cases and similar contexts.

Moreover, case reports, compared to for example, randomized controlled trials or systematic reviews are, with a supportive journal and editorial process, relatively straightforward to write, complete, and publish. Many authors initiate their scholarly writings with case reports and are exhilarated through their initial publications. They are more likely to be motivated to write and publish subsequently if their initial case reports can be published without too many significant hurdles.

CY What are your views on open access publishing?

PP Amongst many transformations in global biomedical journals, one of the major triumphs is the shift towards Open Access. Open access has empowered a much wider readership of global researchers and readers, compared to pure subscription publishing models, as evidenced by the exceedingly high number of open access article downloads. Open access is essential to make science both accessible and inclusive especially for the clinicians and researchers based in LMICs.

Often, in an attempt to access high‐quality research from academically renowned but “pay‐walled” journals, I am faced with a financial hassle which I am unable to deal with and so my academic progress is restricted. As a result, I am personally grateful for the open access transformation, which has allowed equitable access to scientific and clinical information for global researchers and clinicians, especially those from the poorer regions.

CY Do you know friends or colleagues who have not published their research because of article publication charges?

PP I'm acquainted with many fellow colleagues from the South Asian subcontinent, who are reluctant to publish because of APCs. I remember them sharing their experience of settling their APCs by collecting funds from all the coauthors to make the individual impact of the payment less for everyone.

CY What are your views on APC waivers?

PP APC waivers are one of the major factors which have encouraged me, and other early career researchers I know, to pursue publication from LMICs. I have experienced the profound significance of the waiver first‐hand, without which my career would not have been so successful to date.

Clinical Case Reports (CCR) has a relatively high number of early‐career authors, who are new to biomedical publishing and who often lack a secure financial status. The relative financial impact of APCs may be greater on these early‐career authors. In retrospect, I pay my sincere gratitude for the APC waivers that were granted to me, for marching me from Zero through to the 30th publication in the third year of my practice, despite various other challenges.

CY What is it like being an early‐career author?

PP It is exciting and adventurous, but at the same time an arduous journey. As an early‐career author, I'm determined to proceed with passion, compassion, empathy, and integrity and to persevere with my motive to improve global health outcomes in the longer term. Although faced with limitations, every cloud has silver lining, and I have been lucky to have met mentors and editors who have helped to motivate me during challenging times.

CY How else can journals help early‐career authors?

PP Journals can partake a collaborative approach within the editorial process allowing a more liberal approach to formatting submissions, flexibility around deadlines, and collaborative peer review to help encourage early‐career authors, especially those from the LMICs. Moreover, all the entities involved in the publication process need to be aware of the importance of making the publication journey hassle‐free and supportive. As an example, language barriers can sometimes be addressed by a considerate approach to editing, and in‐house language editing services offered publishers. Overall, journals can help early‐career authors by adopting a gentle approach, and by being considerate about those authors' initial submissions. Furthermore, language should not be the determinant factor during preliminary rounds of reviews.

However, in response to this considerate editorial approach, the content and significance of the submissions must be of as high a quality as possible as this is imperative for the quality and impact of the journal. Researchers from LMICs also need to focus on capability building and enhancing their training in professional scientific writing. Moreover, early‐career authors can also gain essential experience by the provision of basic training in publication ethics, and editorial processes through active involvement with the editorial team and process—following the current approach of CCR.

CY What is your advice to other early‐career authors?

PP The early career stages are an exciting phase in one's career, filled with challenges as well as vast opportunities. Early‐career authors need to be persistent and persevere with their writing—starting with publications such as case images, videos and case reports. Rejections and denials are part of the publication process, and early‐stage authors must not be deterred by these issues when they occur. Authors should be focused on capability building, training with language, scholarly writing skills and familiarize themselves with publication ethics. Early‐career authors should try to publish as frequently as possible, which will motivate them and may also help them to acquire further support schemes like scholarships and travel grants further adding to their academic growth.

CY What are your future career and research plans?

PP I'm a full‐time clinical dermatologist based in a government hospital, where I devote most of my time to clinical practice and patient care. In future, I wish to enrich my academic experience, clinical skill and write relevant research that is important in our local context. I have always been fascinated by the editorial process and since the start of my career, I have been serving as an Associate Editor of four international journals, including Clinical Case Reports, as well as being a reviewer of six international journals. In future, I want to be more actively involved in the editorial process and also to pursue practice oriented around high‐quality research. I hope to apply for research grants that would help me conduct studies without having to worry so much about resources and finances.

## 2 CONCLUSION

Publications by authors from LMICs make a crucial contribution to the scientific record, and in doing so are key to the progression of scientific understanding and the advancement of global healthcare. As Kathryn Sharples writes, “The world needs breakthroughs from change‐makers and innovators who never give up and who keep asking questions.”^5^ APC waivers, along with inclusive editorial policies in journals such as CCR, are key to supporting these change‐makers from LMICs.

## AUTHOR CONTRIBUTIONS

**Prajwal Pudasaini:** Conceptualization; validation; visualization; writing – original draft; writing – review and editing. **Charles Young:** Conceptualization; resources; supervision; validation; visualization; writing – original draft; writing – review and editing.

## FUNDING INFORMATION

None declared.

## CONSENT

Written informed consent was obtained from the patient to publish this report in accordance with the journal’s patient consent policy.

## Data Availability

Data sharing not applicable to this article as no datasets were generated or analysed during the current study.
